# Supplementary material for: Orthogonal control of gene expression in plants using synthetic promoters and CRISPR-based transcription factors
Source: Plant Methods. 2022 Mar 29;18:42. doi: 10.1186/s13007-022-00867-1 (PMC8966344; doi:10.1186/s13007-022-00867-1)
Supplement: Supplementary file 1 — Additional file 1: Fig S1: Workflow describing the assembly of single and multiple transcriptional units (TUs) in a plant expression vector; Fig S2: Western blot to analyze the expression of dCas9:VP64 in OCS constructs – OCS1-1 and OCS 1–5. Table S1: List of all genetic parts used for the construction of OCS constructs. Table S2: List of all OCS constructs. Table S3: List of all Addgene plasmids used in this work [file 13007_2022_867_MOESM1_ESM.pdf]

Additional file for

Orthogonal control of gene expression using synthetic promoters and CRISPR-based transcription factors

Shaunak Kar[1,2,#], Yogendra Bordiya[1,4,#], Nestor Rodriguez[1], Junghyun Kim[1], Elizabeth C. Gardner[1,2], Jimmy Gollihar[2], Sibum Sung[1,*] and Andrew D. Ellington[1,2,*]

[1] Department of Molecular Biosciences, University of Texas at Austin, Austin TX, USA
[2] Center for Systems and Synthetic Biology, University of Texas at Austin, Austin, TX, USA
[3] US Army Research Laboratories−South, Austin, Texas, USA
[4] Present address: Life Sciences Solutions group, Thermo Fisher Scientific, Austin, TX, USA

[#] Authors contributed equally
[*] Corresponding authors

Sibum Sung: sbsung@austin.utexas.edu, Andrew D. Ellington: ellingtonlab@gmail.com

Figure S1: Workflow describing the assembly of single and multiple transcriptional units (TUs) in a plant expression vector

Figure S2: Western blot to analyze the expression of dCas9:VP64 in OCS constructs – OCS1-1 and OCS 1-5

Table S1: List of all genetic parts used for the construction of OCS constructs

Table S2: List of all OCS constructs

Table S3: List of all Addgene plasmids used in this work

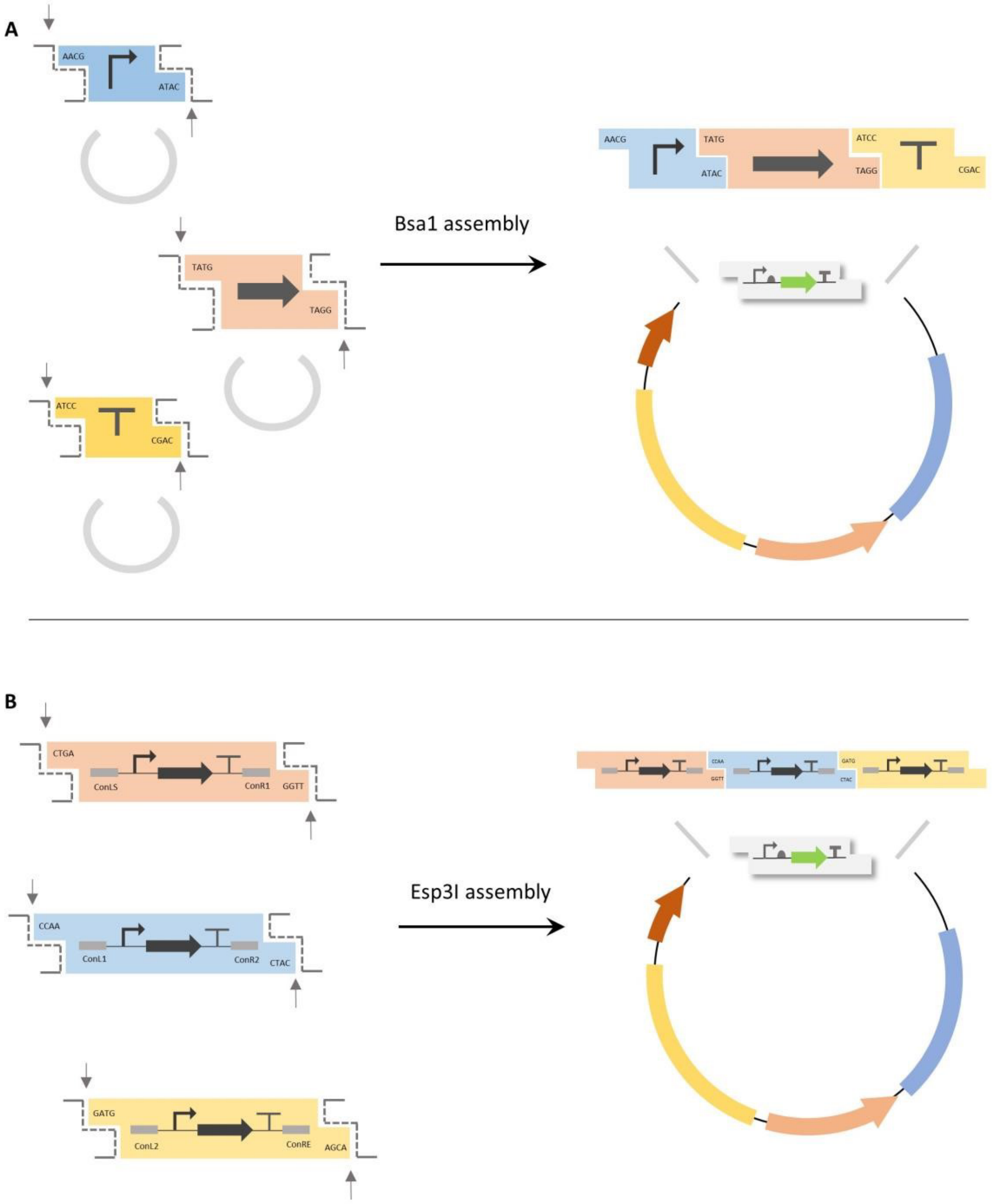

Figure S1: Schematic demonstrating the assembly of single (A) or multiple (B) transcriptional units into a plant expression vector. A single transcription unit consists of a promoter, gene and a terminator parts while for multiple transcriptional units, each TU is flanked by appropriate connector sequences. The arrows depict the restriction sites for Bsa1 (A) and Esp3I (B). B) Schematic showing the assembly of multiple TUs into plant expression vector where each TU is encoded in separate plasmids flanked by appropriate connector sequences.

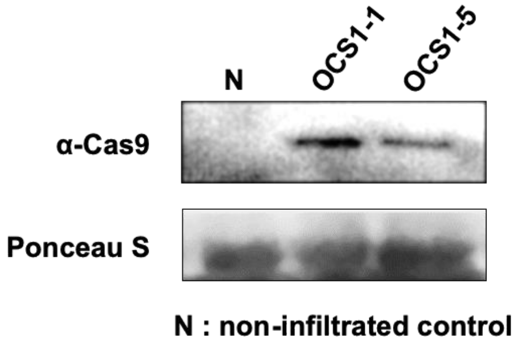

Figure S2: The expression of hdCas9 for both OCS 1-1 and OCS 1-5 was confirmed via Western blot analysis (see Methods).

Table S1: List of genetic elements used.

Highlighted regions in red indicate BsaI recognition sites and the corresponding overhangs are highlighted in blue.

S1.1 Promoters

Name	Description	Sequence
P1	35S promoter	GGTCTCAAACGCGTCAACATGGTGGAGCACGACACTCTGGTCT<br>ACTCCAAAAATGTCAAAGATACAGTCTCAGAAGATCAAAGGGCT<br>ATTGAGACTTTTCAACAAAGGATAATTTCGGGAAACCTCCTCGG<br>ATTCCATTGCCCAGCTATCTGTCACTTCATCGAAAGGACAGTAG<br>AAAAGGAAGGTGGCTCCTACAAATGCCATCATTGCGATAAAGG<br>AAAGGCTATCATTCAAGATCTCTCTGCCGACAGTGGTCCCAAA<br>GATGGACCCCCACCCACGAGGAGCATCGTGGAAAAAGAAGAG<br>GTTCCAACCACGTCTACAAAGCAAGTGGATTGATGTGACATCTC<br>CACTGACGTAAGGGATGACGCACAATCCCACTATCCTTCGCAA<br>GACCCTTCCTCTATATAAGGAAGTTCATTTCATTTGGAGAGGAC<br>ACGCGTTTATTTACAAGAGCGTACGGTTCAATCCCTGCCTCCC<br>CTGTAAAACTACCCTTTGAAAACCTCTCTTTCTTAATCTTTTCTT<br>TGTAATTCCAGATCTATGTGAGACC
P2	Mas promoter	GGTCTCAAACGCGGAGATTTTTCAAATCAGTGCGCAAGACGTG<br>ACGTAAGTATCCGAGTCAGTTTTTATTTTTCTACTAATTTGGTCG<br>TTTATTTCGGCGTGTAGGACATGGCAACCGGGCCTGAATTTCG<br>CGGGTATTCTGTTTCTATTCCAACTTTTTCTTGATCCGCAGCCA<br>TTAACGACTTTTGAATAGATACGCTGACACGCCAAGCCTCGCTA<br>GTCAAAAGTGTACCAAACAACGCTTTACAGCAAGAACGGAATG<br>CGCGTGACGCTCGCGGTGACGCCATTTCGCCTTTTCAGAAATG<br>GATAAATAGCCTTGCTTCCTATTATATCTTCCCAAATTACCAATA<br>CATTACACTAGCATCTGAATTTCATAACCAATCTCGATACACCA<br>AATCGAGATCTATGTGAGACC
P4	EBS promoter	GGTCTCAAACGCATTACGAATTCCCGGGGATCCTCATGATCAA<br>AGGGGGGATGCACTATTTAAGATCCTCATGATCAAAGGGGGGA<br>TGCACTATTTAAGATCCTCATGATCAAAGGGGGGATGCACTATT<br>TAAGATCCTCATGATCAAAGGGGGGATGCACTATTTAAGATCCT<br>CATGATCAAAGGGGGGATGCACTATTTAAGATCCTTCGCAAGA<br>CCCTTCCTCTATATAAGGAAGTTCATTTCATTTGGAGAGGACAG<br>GGTATCAAGCTTGGCACTGGCCGTCGTTTTACAACGTCGTGAC<br>TGGGAAAACCCTGGCGTTACCCAACTTAATCGCCTTGCAGCAC<br>ATCCCCCTTTCGCCAGCTGGCGTAATAGCGAAGAGGCCCGCAC<br>CGATCGCCCTTCCCAACAGTTGCGCAGCCTGAAGCCTAGGGA<br>GGAGTCCACTCTATGTGAGACC

pATF-1	Artificial promoter 1 (3 gRNA repeats)	GGTCTCAAACGCCAAACCACAATTTGCACACCCTGGCATCTCA<br>AACCACAATTTGCACACCCTGGCATCTCAAACCACAATTTGCAC<br>ACCCTGGCATCTCCGCAAGACCCTTCCTCTATATAAGGAAGTTC<br>ATTTCATTTGGAGAGGACACGCGTTTATTTACAAGAGCGTACGG<br>TTCAATCCCTGCCTCCCCTGTAAAACTACCCTTTGAAAACCTCT<br>CTTTCTTAATCTTTTCTTTGTAATTCCAGATCTATGTGAGACC
pATF-3	Artificial promoter 3 (3 gRNA repeats)	GGTCTCAAACGCCTGACTCACAGTCCTATCGAGTGGCATCTCT<br>GACTCACAGTCCTATCGAGTGGCATCTCTGACTCACAGTCCTAT<br>CGAGTGGCATCTCCGCAAGACCCTTCCTCTATATAAGGAAGTT<br>CATTTCATTTGGAGAGGACACGCGTTTATTTACAAGAGCGTACG<br>GTTCAATCCCTGCCTCCCCTGTAAAACTACCCTTTGAAAACCTC<br>TCTTTCTTAATCTTTTCTTTGTAATTCCAGATCTATGTGAGACC
pATF-4	Artificial promoter 4 (3 gRNA repeats)	GGTCTCAAACGCCATTGCGCACCATTCCACTAGTGGCATCTCA<br>TTGCGCACCATTCCACTAGTGGCATCTCATTGCGCACCATTCCA<br>CTAGTGGCATCTCCGCAAGACCCTTCCTCTATATAAGGAAGTTC<br>ATTTCATTTGGAGAGGACACGCGTTTATTTACAAGAGCGTACGG<br>TTCAATCCCTGCCTCCCCTGTAAAACTACCCTTTGAAAACCTCT<br>CTTTCTTAATCTTTTCTTTGTAATTCCAGATCTATGTGAGACC

S1.2: Terminators

Name	Description	Sequence
T1	35S Terminator	GGTCTCAATCCTAACTCGAGCTCTAGCTAGAGTCGATCGACAA<br>GCTCGAGTTTCTCCATAATAATGTGTGAGTAGTTCCCAGATAAG<br>GGAATTAGGGTTCCTATAGGGTTTCGCTCATGTGTTGAGCATAT<br>AAGAAACCCTTAGTATGTATTTGTATTTGTAAAATACTTCTATCA<br>ATAAAATTTCTAATTCCTAAAACCAAAATCCAGTACTAAAATCCA<br>GATGCTGTGAGACC

S1.3 Coding sequences

Name	Description	Sequence
G1	YFP	GGTCTCATATGGTATCCAAAGGAGAAGAATTGTTTACAGGGGTA<br>GTCCCCATATTGGTCGAGCTCGATGGGGATGTAAACGGCACA<br>AGTTCTCAGTGTCTGGCGAAGGCGAAGGGGACGCCACATACG<br>GAAAGTTGACTCTGAAGTTCATCTGCACAACCGGTAAACTACCC<br>GTACCCTGGCCAACACTGGTGACGACCTTTGGGTACGGGCTAC<br>AATGTTTCGCTCGATATCCTGACCACATGAAGCAACACGATTTC<br>TTCAAAAGCGCCATGCCTGAGGGGTATGTACAGGAGCGTACCA<br>TATTTTTCAAAGACGATGGAAACTATAAGACCCGAGCTGAAGTG<br>AAATTTGAAGGAGATACGTTAGTCAATCGAATAGAACTAAAGGG

		AATTGATTTCAAGGAGGATGGGAATATTCTGGGCCATAAGCTG<br>GAGTACAATTACAATAGTCACAACGTCTATATAATGGCAGACAA<br>GCAGAAGAATGGAATAAAGGTTAATTTCAAGATAAGGCATAACA<br>TTGAAGACGGAAGCGTTCAGCTAGCCGATCATTACCAACAGAA<br>TACACCGATAGGTGATGGACCCGTCCTGCTCCCCGACAACCAT<br>TACTTGTCTTACCAGAGTGCACTTTCTAAAGATCCAAATGAGAA<br>AAGAGATCATATGGTTTTATTAGAATTTGTTACCGCAGCCGGCA<br>TAACTTTGGGAATGGACGAACTGTACAAATGAGGATCCTGAGA<br>CC
G2	RFP	GGTCTCATATGAGCGAGCTGATTAAGGAGAACATGCACATGAA<br>GCTGTACATGGAGGGCACCGTGAACAACCACCACTTCAAGTGC<br>ACATCCGAGGGCGAAGGCAAGCCCTACGAGGGCACCCAGACC<br>ATGAGAATCAAGGTGGTCGAGGGCGGCCCTCTCCCCTTCGCC<br>TTCGACATCCTGGCTACCAGCTTCATGTACGGCAGCAGAACCT<br>TCATCAACCACACCCAGGGCATCCCCGACTTCTTTAAGCAGTC<br>CTTCCCTGAGGGCTTCACATGGGAGAGAGTCACCACATACGAA<br>GACGGGGGCGTGCTGACCGCTACCCAGGACACCAGCCTCCAG<br>GACGGCTGCCTCATCTACAACGTCAAGATCAGAGGGGTGAACT<br>TCCCATCCAACGGCCCTGTGATGCAGAAGAAAACACTCGGCTG<br>GGAGGCCAACACCGAGATGCTGTACCCCGCTGACGGCGGCCT<br>GGAAGGCAGAAGCGACATGGCCCTGAAGCTCGTGGGCGGGG<br>GCCACCTGATCTGCAACTTCAAGACCACATACAGATCCAAGAA<br>ACCCGCTAAGAACCTCAAGATGCCCGGCGTCTACTATGTGGAC<br>CACAGACTGGAAAGAATCAAGGAGGCCGACAAAGAAACTTACG<br>TCGAGCAGCATGAGGTTGCTGTGGCCAGATACTGCGACCTCCC<br>TAGCAAACTGGGGCACAAGTGAGGATCCTGAGACC
G10	BFP	GGTCTCATATGAGCGAAGAACTAATCAAGGAAAATATGCACATG<br>AAACTCTACATGGAGGGTACGGTTGACAATCATCATTTCAAATG<br>TACCGCAGAGGGCGAAGGTAAACCCTATGAGGGCACCCAGAC<br>TATGCGAATCAAGGTTGTGGAGGGCGGGCCATTGCCCTTCGCT<br>TTTGACATCTTAGCTACTAGTTTCTTATATGGGAGCAAGACCTTT<br>ATAGATCACACACAGGGTATCCCGGACTTCTTTAAACAGAGCTT<br>TCCAGAAGGGTTCACCTGGGAAAGGGTGACAACCTATGAAGAT<br>GGCGGAGTGCTTACAGCGACACAAGACACCTCCCTACAGGAC<br>GGCACCCTAATATATAATGTCAAGATTCGTGGCGTAGATTTCCT<br>CAGCAATGGCCCTGTTATGCAGAAGAAGACACTTGGATGGGAG<br>GCTTTCACTGAAACCCTCTACCCGGCGGATGGAGGCTTAGAGG<br>GGAGAAACGATATGGCGTTAAAGCTGGTCGGGGGATCACACTT<br>GATCGCGCATGCAAAAACTACGTACAGGTCCAAGAAACCAGCA<br>AAGAATCTCAAGATGCCAGGTGTATACTATGTGGATTACCGACT<br>CGAGCGTATTAAGGAAGCAAACGACGAAACGTACGTCGAACAA<br>CACGAAGTTGCAGTAGCAAGGTATAGCGACCTTCCCTCCAAGC<br>TAGGACATAAGCTGAATGGGAGCGGATAAGGATCCTGAGACC
G17	Luciferase	GGTCTCATATGGAAGATGCAAAGAATATCAAAAAAGGCCCAGC<br>GCCCTTCTACCCATTAGAAGATGGAACCGCAGGAGAGCAACTT<br>CACAAGGCGATGAAACGATATGCTCTTGTCCCGGGAACCATCG<br>CTTTTACGGACGCACACATAGAGGTTAACATTACCTATGCGGAA<br>TATTTTGAAATGTCAGTCAGATTAGCAGAAGCAATGAAACGTTA<br>TGGGCTCAACACTAACCATCGTATTGTTGTATGTAGCGAAAACA<br>GCCTGCAGTTTTTTATGCCGGTGCTCGGTGCGCTGTTCATCGG<br>TGTAGCGGTTGCTCCCGCAAACGACATTTACAACGAGAGAGAA<br>CTGCTTAACAGCATGAATATCAGCCAACCGACCGTCGTGTTTGT

		CTCAAAAAAGGGACTACAAAAAATTCTAAATGTCCAAAAGAAGT<br>TACCTATTATCCAGAAAATTATTATTATGGATAGCAAGACGGATT<br>ATCAAGGATTCCAATCTATGTACACATTTGTTACGAGCCACTTA<br>CCTCCAGGTTTTAACGAATATGATTTTGTGCCTGAGAGCTTTGA<br>TCGAGATAAGACCATCGCGTTAATTATGAATAGTTCCGGCTCTA<br>CGGGGCTCCCAAAGGGAGTCGCACTACCACATCGAACTGCGT<br>GCGTTAGATTTTCACATGCCAGAGATCCTATCTTCGGGAATCAG<br>ATTATTCCGGACACTGCAATACTGAGTGTGGTTCCGTTTCATCA<br>CGGGTTCGGGATGTTCACGACACTCGGTTACCTCATATGCGGA<br>TTTCGTGTGGTGCTGATGTATAGGTTTGAAGAAGAGTTGTTCCT<br>AAGATCCTTGCAGGATTACAAAATTCAGTCCGCCCTGTTAGTTC<br>CTACCTTATTTTCCTTTTTCGCCAAGTCAACGTTAATTGATAAAT<br>ATGACCTATCCAACCTCCACGAAATTGCCAGTGGTGGGGCCCC<br>CTTGTCCAAAGAAGTTGGTGAGGCAGTCGCTAAGAGGTTCCAC<br>CTGCCGGGTATCCGTCAAGGCTACGGACTTACCGAAACAACCT<br>CCGCTATTCTTATTACACCTGAAGGCGATGATAAGCCGGGTGC<br>TGTCGGTAAGGTGGTGCCTTTTTTTGAGGCGAAGGTAGTGGAC<br>TTAGATACTGGCAAGACGCTCGGAGTTAATCAACGAGGCGAGC<br>TCTGTGTCCGTGGTCCCATGATAATGAGCGGATACGTCAACAA<br>TCCTGAGGCAACCAACGCATTAATTGATAAAGACGGCTGGTTG<br>CATAGCGGGGATATCGCGTATTGGGATGAGGACGAACACTTCT<br>TTATAGTCGATAGGTTAAAGTCACTTATTAAGTATAAAGGTTATC<br>AAGTCGCTCCCGCCGAACTGGAGAGTATTCTTCTTCAGCATCC<br>GAATATCTTTGACGCCGGTGTTGCAGGTCTTCCTGACGACGAC<br>GCCGGAGAACTACCTGCAGCGGTCGTGGTGCTCGAACATGGA<br>AAGACAATGACCGAGAAGGAGATTGTAGATTACGTAGCTTCAC<br>AGGTCACCACCGCTAAAAAACTAAGAGGTGGTGTTGTCTTCGT<br>AGATGAAGTGCCTAAAGGACTTACCGGTAAACTCGACGCCAGG<br>AAGATAAGAGAAATCCTGATCAAAGCGAAGAAGGGTGGTAAGT<br>CTAAGCTGTAAGGATCCTGAGACC
G15	dCas9:VP64	GGTCTCATATGCCCAAGAAGAAGAGGAAGGTGGACAAGAAGTA<br>CTCCATTGGGCTCGCTATCGGCACAAACAGCGTCGGCTGGGC<br>CGTCATTACGGACGAGTACAAGGTGCCGAGCAAAAAATTCAAA<br>GTTCTGGGCAATACCGATCGCCACAGCATAAAGAAGAACCTCA<br>TTGGCGCCCTCCTGTTCGACTCCGGGGAAACGGCCGAAGCCA<br>CGCGGCTCAAAAGAACAGCACGGCGCAGATATACCCGCAGAA<br>AGAATCGGATCTGCTACCTGCAGGAGATCTTTAGTAATGAGAT<br>GGCTAAGGTGGATGACTCTTTCTTCCATAGGCTGGAGGAGTCC<br>TTTTTGGTGGAGGAGGATAAAAAGCACGAGCGCCACCCAATCT<br>TTGGCAATATCGTGGACGAGGTGGCGTACCATGAAAAGTACCC<br>AACCATATATCATCTGAGGAAGAAGCTTGTAGACAGTACTGATA<br>AGGCTGACTTGCGGTTGATCTATCTCGCGCTGGCGCATATGAT<br>CAAATTTCGGGGACACTTCCTCATCGAGGGGGACCTGAACCCA<br>GACAACAGCGATGTCGACAAACTCTTTATCCAACTGGTTCAGAC<br>TTACAATCAGCTTTTCGAAGAGAACCCGATCAACGCATCCGGA<br>GTTGACGCCAAAGCAATCCTGAGCGCTAGGCTGTCCAAATCCC<br>GGCGGCTCGAAAACCTCATCGCACAGCTCCCTGGGGAGAAGA<br>AGAACGGCCTGTTTGGTAATCTTATCGCCCTGTCACTCGGGCT<br>GACCCCCAACTTTAAATCTAACTTCGACCTGGCCGAAGATGCC<br>AAGCTTCAACTGAGCAAAGACACCTACGATGATGATCTCGACA<br>ATCTGCTGGCCCAGATCGGCGACCAGTACGCAGACCTTTTTTT<br>GGCGGCAAAGAACCTGTCAGACGCCATTCTGCTGAGTGATATT<br>CTGCGAGTGAACACGGAGATCACCAAAGCTCCGCTGAGCGCT

		AGTATGATCAAGCGCTATGATGAGCACCACCAAGACTTGACTTT<br>GCTGAAGGCCCTTGTCAGACAGCAACTGCCTGAGAAGTACAAG<br>GAAATTTTCTTCGATCAGTCTAAAAATGGCTACGCCGGATACAT<br>TGACGGCGGAGCAAGCCAGGAGGAATTTTACAAATTTATTAAG<br>CCCATCTTGGAAAAAATGGACGGCACCGAGGAGCTGCTGGT<br>AAAGCTTAACAGAGAAGATCTGTTGCGCAAACAGCGCACTTTC<br>GACAATGGAAGCATCCCCCACCAGATTCACCTGGGCGAACTGC<br>ACGCTATCCTCAGGCGGCAAGAGGATTTCTACCCCTTTTTGAAA<br>GATAACAGGGAAAAGATTGAGAAAATCCTCACATTTCGGATACC<br>CTACTATGTAGGCCCCCTCGCCCGGGGAAATTCCAGATTCGCG<br>TGGATGACTCGCAAATCAGAAGAGACTATCACTCCCTGGAACTT<br>CGAGGAAGTCGTGGATAAGGGGGCCTCTGCCCAGTCCTTCATC<br>GAAAGGATGACTAACTTTGATAAAAATCTGCCTAACGAAAAGGT<br>GCTTCCTAAACACTCTCTGCTGTACGAGTACTTCACAGTTTATA<br>ACGAGCTCACCAAGGTCAAATACGTCACAGAAGGGATGAGAAA<br>GCCAGCATTCCTGTCTGGAGAGCAGAAGAAAGCTATCGTGGAC<br>CTCCTCTTCAAGACGAACCGGAAAGTTACCGTGAAACAGCTCA<br>AAGAAGATTATTTCAAAAAGATTGAATGTTTCGACTCTGTTGAAA<br>TCAGCGGAGTGGAGGATCGCTTCAACGCATCCCTGGGAACGTA<br>TCACGATCTCCTGAAAATCATTAAAGACAAGGACTTCCTGGACA<br>ATGAGGAGAACGAGGACATTCTTGAGGACATTGTCCTCACCCT<br>TACGTTGTTTGAAGATAGGGAGATGATTGAAGAACGCTTGAAAA<br>CTTACGCTCATCTCTTCGACGACAAAGTCATGAAACAGCTCAAG<br>AGGCGCCGATATACAGGATGGGGGCGGCTGTCAAGAAAACTG<br>ATCAATGGGATCCGAGACAAGCAGAGTGGAAAGACAATCCTGG<br>ATTTTCTTAAGTCCGATGGATTTGCCAACCGGAACTTCATGCAG<br>TTGATCCATGATGACTCTCTCACCTTTAAGGAGGACATCCAGAA<br>AGCACAAGTTTCTGGCCAGGGGGACAGTCTCCACGAGCACATC<br>GCTAATCTTGCAGGTAGCCCAGCTATCAAAAAGGGAATACTGC<br>AGACCGTTAAGGTCGTGGATGAACTCGTCAAAGTAATGGGAAG<br>GCATAAGCCCGAGAATATCGTTATCGAGATGGCCCGAGAGAAC<br>CAAACTACCCAGAAGGGACAGAAGAACAGTAGGGAAAGGATGA<br>AGAGGATTGAAGAGGGTATAAAAGAACTGGGGTCCCAAATCCT<br>TAAGGAACACCCAGTTGAAAACACCCAGCTTCAGAATGAGAAG<br>CTCTACCTGTACTACCTGCAGAACGGCAGGGACATGTACGTGG<br>ATCAGGAACTGGACATCAATCGGCTCTCCGACTACGACGTGGA<br>TGCCATCGTGCCCCAGTCTTTTCTCAAAGATGATTCTATTGATA<br>ATAAAGTGTTGACAAGATCCGATAAAAATAGAGGGAAGAGTGAT<br>AACGTCCCCTCAGAAGAAGTTGTCAAGAAAATGAAAAATTATTG<br>GCGGCAGCTGCTGAACGCCAAACTGATCACACAACGGAAGTTC<br>GATAATCTGACTAAGGCTGAACGAGGTGGCCTGTCTGAGTTGG<br>ATAAAGCCGGCTTCATCAAAAGGCAGCTTGTTGAGACACGCCA<br>GATCACCAAGCACGTGGCCCAAATTCTCGATTCACGCATGAAC<br>ACCAAGTACGATGAAAATGACAAACTGATTCGAGAGGTGAAAG<br>TTATTACTCTGAAGTCTAAGCTGGTTTCAGATTTCAGAAAGGAC<br>TTTCAGTTTTATAAGGTGAGAGAGATCAACAATTACCACCATGC<br>GCATGATGCCTACCTGAATGCAGTGGTAGGCACTGCACTTATC<br>AAAAAATATCCCAAGCTTGAATCTGAATTTGTTTACGGAGACTAT<br>AAAGTGTACGATGTTAGGAAAATGATCGCAAAGTCTGAGCAGG<br>AAATAGGCAAGGCCACCGCTAAGTACTTCTTTTACAGCAATATT<br>ATGAATTTTTTCAAGACCGAGATTACACTGGCCAATGGAGAGAT<br>TCGGAAGCGACCACTTATCGAAACAAACGGAGAAACAGGAGAA<br>ATCGTGTGGGACAAGGGTAGGGATTTCGCGACAGTCCGGAAG

		GTCCTGTCCATGCCGCAGGTGAACATCGTTAAAAAGACCGAAG TACAGACCGGAGGCTTCTCCAAGGAAAGTATCCTCCCGAAAAG GAACAGCGACAAGCTGATCGCACGCAAAAAAGATTGGGACCCC AAGAAATACGGCGGATTCGATTCTCCTACAGTCGCTTACAGTGT ACTGGTTGTGGCCAAAGTGGAGAAAGGGAAGTCTAAAAAACTC AAAAGCGTCAAGGAACTGCTGGGCATCACAATCATGGAGCGAT CAAGCTTCGAAAAAAACCCCATCGACTTTCTCGAGGCGAAAGG ATATAAAGAGGTCAAAAAAGACCTCATCATTAAGCTTCCCAAGT ACTCTCTCTTTGAGCTTGAAAACGGCCGGAAACGAATGCTCGC TAGTGCGGGCGAGCTGCAGAAAGGTAACGAGCTGGCACTGCC CTCTAAATACGTTAATTTCTTGTATCTGGCCAGCCACTATGAAAA GCTCAAAGGATCTCCCGAAGATAATGAGCAGAAGCAGCTGTTC GTGGAACAACACAAACACTACCTTGATGAGATCATCGAGCAAAT AAGCGAATTCTCCAAAAGAGTGATCCTCGCCGACGCTAACCTC GATAAGGTGCTTTCTGCTTACAATAAGCACAGGGATAAGCCCAT CAGGGAGCAGGCAGAAAACATTATCCACTTGTTTACTCTGACCA ACTTGGGCGCGCCTGCAGCCTTCAAGTACTTCGACACCACCAT AGACAGAAAGCGGTACACCTCTACAAAGGAGGTCCTGGACGCC ACACTGATTCATCAGTCAATTACGGGGCTCTATGAAACAAGAAT CGACCTCTCTCAGCTCGGTGGAGACAGCAGGGCTGATTCGGA CCCAAAAAAGAAGCGTAAGGTCGATCCCAAGAAGAAGAGAAAG GTAGATCCTAAGAAGAAGAGAAAGGTAGACGCATTGGATGACT TCGACTTAGACATGCTTGGGTCAGATGCTTTGGACGACTTTGAC CTCGACATGTTGGGTTCTGACGCGCTAGATGACTTCGACTTGG ACATGTTGGGCTCCGACGCTTTGGACGACTTTGATCTGGACAT GTTATGAGGATCCTGAGACC

S1.4: gRNA expression cassettes

Name	Description	Sequence
U6-gRNA-1	U6 promoter driving gRNA-1 expression	GGTCTCAAACGTTCGACGTAAAGCCTGTAGAAGAGGTTTCTAG CGAACGACACGAGTTTGAGCCTCATGAAGCTTCGTTGAACAAC GGAAACTCGACTTGCCTTCCGCACAATACATCATTTCTTCTTAG CTTTTTTTCTTCTTCTTCGTTCATACAGTTTTTTTTTGTTTATCAG CTTACATTTTCTTGAACCGTAGCTTTCGTTTTCTTCTTTTTAACTT TCCATTCGGAGTTTTTGTATCTTGTTTCATAGTTTGTCCCAGGAT TAGAATGATTAGGCATCGAACCTTCAAGAATTTGATTGAATAAA ACATCTTCATTCTTAAGATATGAAGATAATCTTCAAAAGGCCCCT GGGAATCTGAAAGAAGAGAAGCAGGCCCATTTATATGGGAAAG AACAATAGTATTTCTTATATAGGCCCATTTAAGTTGAAAACAATC TTCAAAAGTCCCACATCGCTTAGATAAGAAAACGAAGCTGAGTT TATATACAGCTAGAGTCGAAGTAGTGATTAAACCACAATTTGCA CACCCGTTTTAGAGCTAGAAATAGCAAGTTAAAATAAGGCTAGT CCGTTATCAACTTGAAAAAGTGGCACCGAGTCGGTGCTTTTTTT GCAAAATTTTCCAGATCGATTTCTTCTTCCTCTGTTCTTCGGCG TTCAATTTCTGGGTTTTTCTCTTCGTTTTCTGTAACTGAAACCTA AAATTTGACCTAAAAAAAATCTCAAATAATATGATTCAGTGGTTT TGTACTTTTCAGTTAGTTGAGTTTTGCAGTTCCGATGAGATAAA CCAATAACTTTGCTTAGATCTAATTCATTCCGTTACACCTCTGAT GGAGATGGAAGGTTCTTAATAATGATGCCATTTTTTGGGTAATA ATTTTGAATTAGAATCAAGGGTATAAGATTCATAATTAACA

		TCACTTAAGCAAAGTTCGTAATATACGACCACAGGATATAATTTTTGGTACGCTGTGAGACC
U6-gRNA-3	U6 promoter driving gRNA-3 expression	GGTCTCAAACGTTCGACGTAAAGCCTGTAGAAGAGGTTTCTAGCGAACGACACGAGTTTGAGCCTCATGAAGCTTCGTTGAACAACGGAAACTCGACTTGCCTTCCGCACAATACATCATTTCTTCTTAGCTTTTTTTCTTCTTCTTCGTTCATACAGTTTTTTTTTGTTTATCAGCTTACATTTTCTTGAACCGTAGCTTTCGTTTTCTTCTTTTTAACTTTCCATTCGGAGTTTTTGTATCTTGTTTCATAGTTTGTCCCAGGATTAGAATGATTAGGCATCGAACCTTCAAGAATTTGATTGAATAAAACATCTTCATTCTTAAGATATGAAGATAATCTTCAAAAGGCCCCTGGGAATCTGAAAGAAGAGAAGCAGGCCCATTTATATGGGAAAGAACAATAGTATTTCTTATATAGGCCCATTTAAGTTGAAAACAATCTTCAAAAGTCCCACATCGCTTAGATAAGAAAACGAAGCTGAGTTTATATACAGCTAGAGTCGAAGTAGTGATTTGACTCACAGTCCTATCGAGGTTTTAGAGCTAGAAATAGCAAGTTAAAATAAGGCTAGTCCGTTATCAACTTGAAAAAGTGGCACCGAGTCGGTGCTTTTTTTGCAAAATTTTCCAGATCGATTTCTTCTTCCTCTGTTCTTCGGCGTTCAATTTCTGGGTTTTTCTCTTCGTTTTCTGTAACTGAAACCTAAAATTTGACCTAAAAAAAATCTCAAATAATATGATTCAGTGGTTTTGTACTTTTCAGTTAGTTGAGTTTTGCAGTTCCGATGAGATAAACCAATAACTTTGCTTAGATCTAATTCATTCCGTTACACCTCTGATGGAGATGGAAGGTTCTTAATAATGATGCCATTTTTTGGGTAATAATTTTGAATTAGAATCAAGGGTATAAGATTCATAATTAACATCACTTAAGCAAAGTTCGTAATATACGACCACAGGATATAATTTTTGGTACGCTGTGAGACC
U6-gRNA-4	U6 promoter driving gRNA-4 expression	GGTCTCAAACGTTCGACGTAAAGCCTGTAGAAGAGGTTTCTAGCGAACGACACGAGTTTGAGCCTCATGAAGCTTCGTTGAACAACGGAAACTCGACTTGCCTTCCGCACAATACATCATTTCTTCTTAGCTTTTTTTCTTCTTCTTCGTTCATACAGTTTTTTTTTGTTTATCAGCTTACATTTTCTTGAACCGTAGCTTTCGTTTTCTTCTTTTTAACTTTCCATTCGGAGTTTTTGTATCTTGTTTCATAGTTTGTCCCAGGATTAGAATGATTAGGCATCGAACCTTCAAGAATTTGATTGAATAAAACATCTTCATTCTTAAGATATGAAGATAATCTTCAAAAGGCCCCTGGGAATCTGAAAGAAGAGAAGCAGGCCCATTTATATGGGAAAGAACAATAGTATTTCTTATATAGGCCCATTTAAGTTGAAAACAATCTTCAAAAGTCCCACATCGCTTAGATAAGAAAACGAAGCTGAGTTTATATACAGCTAGAGTCGAAGTAGTGATTATTGCGCACCATTCCACTAGGTTTTAGAGCTAGAAATAGCAAGTTAAAATAAGGCTAGTCCGTTATCAACTTGAAAAAGTGGCACCGAGTCGGTGCTTTTTTTGCAAAATTTTCCAGATCGATTTCTTCTTCCTCTGTTCTTCGGCGTTCAATTTCTGGGTTTTTCTCTTCGTTTTCTGTAACTGAAACCTAAAATTTGACCTAAAAAAAATCTCAAATAATATGATTCAGTGGTTTTGTACTTTTCAGTTAGTTGAGTTTTGCAGTTCCGATGAGATAAACCAATAACTTTGCTTAGATCTAATTCATTCCGTTACACCTCTGATGGAGATGGAAGGTTCTTAATAATGATGCCATTTTTTGGGTAATAATTTTGAATTAGAATCAAGGGTATAAGATTCATAATTAACATCACTTAAGCAAAGTTCGTAATATACGACCACAGGATATAATTTTTGGTACGCTGTGAGACC

HHR-gRNA-1	gRNA-1 expression flanked by 5' HHR and 3' HDV	GGTCTCATATGCGACTACTGATGAGTCCGTGAGGACGAAACGA<br>GTAAGCTCGTCAAACCACAATTTGCACACCCGTTTTAGAGCTAG<br>AAATAGCAAGTTAAAATAAGGCTAGTCCGTTATCAACTTGAAAA<br>AGTGGCACCGAGTCGGTGCTTTTGGCCGGCATGGTCCCAGCC<br>TCCTCGCTGGCGCCGGCTGGGCAACATGCTTCGGCATGGCGA<br>ATGGGACGGATCCTGAGACC
HHR-gRNA-3	gRNA-3 expression flanked by 5' HHR and 3' HDV	GGTCTCATATGGAGTCACTGATGAGTCCGTGAGGACGAAACGA<br>GTAAGCTCGTCTGACTCACAGTCCTATCGAGGTTTTAGAGCTA<br>GAAATAGCAAGTTAAAATAAGGCTAGTCCGTTATCAACTTGAAA<br>AAGTGGCACCGAGTCGGTGCTTTTGGCCGGCATGGTCCCAGC<br>CTCCTCGCTGGCGCCGGCTGGGCAACATGCTTCGGCATGGCG<br>AATGGGACGGATCCTGAGACC
HHR-gRNA-4	gRNA-4 expression flanked by 5' HHR and 3' HDV	GGTCTCATATGCGCAATCTGATGAGTCCGTGAGGACGAAACGA<br>GTAAGCTCGTCATTGCGCACCATTCCACTAGGTTTTAGAGCTAG<br>AAATAGCAAGTTAAAATAAGGCTAGTCCGTTATCAACTTGAAAA<br>AGTGGCACCGAGTCGGTGCTTTTGGCCGGCATGGTCCCAGCC<br>TCCTCGCTGGCGCCGGCTGGGCAACATGCTTCGGCATGGCGA<br>ATGGGACGGATCCTGAGACC

S1.5: Plant expression vector backbone elements

Name	Description	Sequence
Pnos	Nos promoter driving the plant resistance cassette	AGCGGAGAATTAAGGGAGTCACGTTATGACCCCCGCCGATG<br>ACGCGGGACAAGCCGTTTTACGTTTGGAACTGACAGAACCG<br>CAACGTTGAAGGAGCCACTCAGCCGCGGGTTTCTGGAGTTTA<br>ATGAGCTAAGCACATACGTCAGAAACCATTATTGCGCGTTCA<br>AAAGTCGCCTAAGGTCACTATCAGCTAGCAAATATTTCTTGTC<br>AAAAATGCTCCACTGACGTTCCATAAATTCCCCTCGGTATCCA<br>ATTAGAGTCTCATATTCACTCTCAATCCAAATAATCTGCACCG<br>GATCT
KanR (Plant)	Kanamycin coding sequence (plant)	ATGATTGAACAAGATGGATTGCACGCAGGTTCTCCGGCCGCT<br>TGGGTGGAGAGGCTATTCGGCTATGACTGGGCACAACAGAC<br>AATCGGCTGCTCTGATGCCGCCGTGTTCCGGCTGTCAGCGC<br>AGGGGCGCCCGGTTCTTTTTGTCAAGACCGACCTGTCCGGT<br>GCCCTGAATGAACTCCAGGACGAGGCAGCGCGGCTATCGTG<br>GCTGGCCACGACGGGCGTTCCTTGCGCAGCTGTGCTCGACG<br>TTGTCACTGAAGCGGGAAGGGACTGGCTGCTATTGGGCGAA<br>GTGCCGGGGCAGGATCTCCTGTCATCTCACCTTGCTCCTGC<br>CGAGAAAGTATCCATCATGGCTGATGCAATGCGGCGGCTGC<br>ATACGCTTGATCCGGCTACCTGCCCATTCGACCACCAAGCGA<br>AACATCGCATCGAGCGAGCACGTACTCGGATGGAAGCCGGT<br>CTTGTCGATCAGGATGATCTGGACGAAGAGCATCAGGGGCT<br>CGCGCCAGCCGAACTGTTCGCCAGGCTCAAGGCGCGTATGC<br>CCGACGGCGAGGATCTCGTCGTGACTCATGGCGATGCCTGC<br>TTGCCGAATATCATGGTGGAAAATGGCCGCTTTTCTGGATTC<br>ATCGACTGTGGCCGGCTGGGTGTGGCGGACCGCTATCAGGA<br>CATAGCGTTGGCTACCCGTGATATTGCTGAAGAGCTTGGCGG

		CGAATGGGCTGACCGCTTCCTCGTGCTTTACGGTATCGCCG CTCCCGATTCGCAGCGCATCGCCTTCTATCGCCTTCTTGACG AGTTCTTC
Tnos	Nos terminator	CTAGAGTCAAGCAGATCGTTCAAACATTTGGCAATAAAGTTTC TTAAGATTGAATCCTGTTGCCGGTCTTGCGATGATTATCATAT AATTTCTGTTGAATTACGTTAAGCATGTAATAATTAACATGTAA TGCATGACGTTATTTATGAGATGGGTTTTTATGATTAGAGTCC CGCAATTATACATTTAATACGCGATAGAAAACAAAATATAGCG CGCAAACTAGGATAAATTATCGCGCGCGGTGTCATCTATGTT ACTAGATCGA
pVS1 replicon	OriV; RepA ; StaA for propagation in Agrobacterium	CGTGCGGCTGCATGAAATCCTGGCCGGTTTGTCTGATGCCAA GCTGGCGGCCTGGCCGGCCAGCTTGGCCGCTGAAGAAACC GAGCGCCGCCGTCTAAAAAGGTGATGTGTATTTGAGTAAAAC AGCTTGCGTCATGCGGTCGCTGCGTATATGATGCGATGAGTA AATAAACAAATACGCAAGGGGAACGCATGAAGGTTATCGCTG TACTTAACCAGAAAGGCGGGTCAGGCAAGACGACCATCGCA ACCCATCTAGCCCGCGCCCTGCAACTCGCCGGGGCCGATGT TCTGTTAGTCGATTCCGATCCCCAGGGCAGTGCCCGCGATTG GGCGGCCGTGCGGGAAGATCAACCGCTAACCGTTGTCGGCA TCGACCGCCCGACGATTGACCGCGACGTGAAGGCCATCGGC CGGCGCGACTTCGTAGTGATCGACGGAGCGCCCCAGGCGG CGGACTTGGCTGTGTCCGCGATCAAGGCAGCCGACTTCGTG CTGATTCCGGTGCAGCCAAGCCCTTACGACATATGGGCCAC CGCCGACCTGGTGGAGCTGGTTAAGCAGCGCATTGAGGTCA CGGATGGAAGGCTACAAGCGGCCTTTGTCGTGTCGCGGGCG ATCAAAGGCACGCGCATCGGCGGTGAGGTTGCCGAGGCGCT GGCCGGGTACGAGCTGCCCATTCTTGAGTCCCGTATCACGC AGCGCGTGAGCTACCCAGGCACTGCCGCCGCCGGCACAAC CGTTCTTGAATCAGAACCCGAGGGCGACGCTGCCCGCGAGG TCCAGGCGCTGGCCGCTGAAATTAAATCAAAACTCATTTGAG TTAATGAGGTAAAGAGAAAATGAGCAAAAGCACAAACACGCT AAGTGCCGGCCGTCCGAGCGCACGCAGCAGCAAGGCTGCA ACGTTGGCCAGCCTGGCAGACACGCCAGCCATGAAGCGGGT CAACTTTCAGTTGCCGGCGGAGGATCACACCAAGCTGAAGAT GTACGCGGTACGCCAAGGCAAGACCATTACCGAGCTGCTAT CTGAATACATCGCGCAGCTACCAGAGTAAATGAGCAAATGAA TAAATGAGTAGATGAATTTTAGCGGCTAAAGGAGGCGGCATG GAAAATCAAGAACAACCAGGCACCGACGCCGTGGAATGCCC CATGTGTGGAGGAACGGGCGGTTGGCCAGGCGTAAGCGGC TGGGTTGTCTGCCGGCCCTGCAATGGCACTGGAACCCCCAA GCCCGAGGAATCGGCGTGACGGTCGCAAACCATCCGGCCC GGTACAAATCGGCGCGGCGCTGGGTGATGACCTGGTGGAGA AGTTGAAGGCCGCGCAGGCCGCCCAGCGGCAACGCATCGA GGCAGAAGCACGCCCCGGTGAATCGTGGCAAGCGGCCGCT GATCGAATCCGCAAAGAATCCCGGCAACCGCCGGCAGCCGG TGCGCCGTCGATTAGGAAGCCGCCCAAGGGCGACGAGCAAC CAGATTTTTTCGTTCCGATGCTCTATGACGTGGGCACCCGCG ATAGTCGCAGCATCATGGACGTGGCCGTTTTCCGTCTGTCGA AGCGTGACCGACGAGCTGGCGAGGTGATCCGCTACGAGCTT CCAGACGGGCACGTAGAGGTTTCCGCAGGGCCGGCCGGCA TGGCCAGTGTGTGGGATTACGACCTGGTACTGATGGCGGTTT CCCATCTAACCGAATCCATGAACCGATACCGGGAAGGGAAG GGAGACAAGCCCGGCCGCGTGTTCCGTCCACACGTTGCGGA

		CGTACTCAAGTTCTGCCGGCGAGCCGATGGCGGAAAGCAGA AAGACGACCTGGTAGAAACCTGCATTCGGTTAAACACCACGC ACGTTGCCATGCAGCGTACGAAGAAGGCCAAGAACGGCCGC CTGGTGACGGTATCCGAGGGTGAAGCCTTGATTAGCCGCTA CAAGATCGTAAAGAGCGAAACCGGGCGGCCGGAGTACATCG AGATCGAGCTAGCTGATTGGATGTACCGCGAGATCACAGAA GGCAAGAACCCGGACGTGCTGACGGTTCACCCCGATTACTTT TTGATCGATCCCGGCATCGGCCGTTTTCTCTACCGCCTGGCA CGCCGCGCCGCAGGCAAGGCAGAAGCCAGATGGTTGTTCAA GACGATCTACGAACGCAGTGGCAGCGCCGGAGAGTTCAAGA AGTTCTGTTTCACCGTGCGCAAGCTGATCGGGTCAAATGACC TGCCGGAGTACGATTTGAAGGAGGAGGCGGGGCAGGCTGG CCCGATCCTAGTCATGCGCTACCGCAACCTGATCGAGGGCG AAGCATCCGCCGGTTCCTAATGTACGGAGCAGATGCTAGGG CAAATTGCCCTAGCAGGGGAAAAAGGTCGAAAAAGCTTCTTT CCTGTGGATAGCACGTACATTGGGAACCCAAAGCCGTACATT GGGAACCGGAACCCGTACATTGGGAACCCAAAGCCGTACAT TGGGAACCGGTCACACATGTAAGTGACTGATATAAAAGAGAA AAAAGGCGATTTTTCCGCCTAAAACTCTTTAAAACTTATTAAA ACTCTTAAAACCCGCCTGGCCTGTGCATAACTGTCTGGCCAG CGCACAGCCGAAGAGCTGCAAAAAGCGCCTACCCTTCGGTC GCTGCGCTCCCTACGCCCCGCCGCTTCGCGTCGGCCTATCG CGGCCGCTGGCCGCTCAAAAATGGCTGGCCTACGGCCAGG CAATCTACCAGGGCGCGGACAAGCCGCGCCGTCGCCACTCG ACCGCCGGCGCCCACATCAAGGCACC
pMB1 origin	Origin of replication for propagation in E.coli	AAAGGATCTTCCTGAGATCCTTTTTTTCTGCGCGTAATCTGCT GCTTGCAAACAAAAAAACCACCGCTACCAGCGGTGGTTTGTT TGCCGGATCAAGAGCTACCAACTCTTTTTCCGAAGGTAACTG GCTTCAGCAGAGCGCAGATACCAAATACTGTCCTTCTAGTGT AGCCGTAGTTAGGCCACCACTTCAAGAACTCTGTAGCACCGC CTACATACCTCGCTCTGCTAATCCTGTTACCAGTGGCTGCTG CCAGTGGCGATAAGTCGTGTCTTACCGGGTTGGACTCAAGAC GATAGTTACCGGATAAGGCGCAGCGGTCGGGCTGAACGGG GGGTTCGTGCACACAGCCCAGCTTGGAGCGAACGACCTACA CCGAACTGAGATACCTACAGCGTGAGCTATGAGAAAGCGCC ACGCTTCCCGAAGGGAGAAAGGCGGACAGGTATCCGGTAAG CGGCAGGGTCGGAACAGGAGAGCGCACGAGGGAGCTTCCA GGGGGAAACGCCTGGTATCTTTATAGTCCTGTCGGGTTTCGC CACCTCTGACTTGAGCGTCGATTTTTGTGATGCTCGTCAGGG GGGCGGAGCCTATGGAAAAACGCCAGCAACGCG
KanR (bacterial)	Kanamycin expression cassette for bacterial selection	GCCAATTCGTGCGCGGAACCCCTATTTGTTTATTTTTCTAAAT ACATTCAAATATGTATCCGCTCATGAGACAATAACCCTGATAA ATGCTTCAATAATATTGAAAAAGGAAGAGTATGGCTAAAATGA GAATATCACCGGAATTGAAAAAACTGATCGAAAAATACCGCT GCGTAAAAGATACGGAAGGAATGTCTCCTGCTAAGGTATATA AGCTGGTGGGAGAAAATGAAAACCTATATTTAAAAATGACGG ACAGCCGGTATAAAGGGACCACCTATGATGTGGAACGGGAA AAGGACATGATGCTATGGCTGGAAGGAAAGCTGCCTGTTCCA AAGGTCCTGCACTTTGAACGGCATGATGGCTGGAGCAATCTG CTCATGAGTGAGGCCGATGGCGTCCTTTGCTCGGAAGAGTAT GAAGATGAACAAAGCCCTGAAAAGATTATCGAGCTGTATGCG GAGTGCATCAGGCTCTTTCACTCCATCGACATATCGGATTGT CCCTATACGAATAGCTTAGACAGCCGCTTAGCCGAATTGGAT

		TACTTACTGAATAACGATCTGGCCGATGTGGATTGCGAAAAC<br>TGGGAAGAGGACACTCCATTTAAAGATCCGCGCGAGCTGTAT<br>GATTTTTTAAAGACGGAAAAGCCCGAAGAGGAACTTGTCTTTT<br>CCCACGGCGACCTGGGAGACAGCAACATCTTTGTGAAAGAT<br>GGCAAAGTAAGTGGCTTTATTGATCTTGGGAGAAGCGGCAG<br>GGCGGACAAGTGGTATGACATTGCCTTCTGCGTCCGGTCGA<br>TCAGGGAGGATATCGGGGAAGAACAGTATGTCGAGCTATTTT<br>TTGACTTACTGGGGATCAAGCCTGATTGGGAGAAAATAAAAT<br>ATTATATTTTACTGGATGAATTGTTTTAGCTGTCAGACCAAGTT<br>TACTCATATATACTTTAGATTGATTTAAAACTTCATTTTTAATTT<br>AAAAGGATCTAGGTGAAGATCCTTTTTGATAATC
LB	Left border repeat	CTGATGGGCTGCCTGTATCGAGTGGTGATTTTGTGCCGAGCT<br>GCCGGTCGGGGAGCTGTTGGCTGGCTGGTGGCAGGATATAT<br>TGTGGTGTAAACAAATTGACGCTTAGACAACTTAATAACACAT<br>TGCGGACGTTTTTAATGTACTG
RB	Right border repeat	TGGTTGGCACATACAAATGGACGAACGGATAAACCTTTTCAC<br>GCCCTTTTAAATATCCGATTATTCTAATAAACGCTCTTTTCTCT<br>TAGGTTTACCCGCCAATATATCCTGTCAAACACTGATAGTTT
GFP DO	GFP expression cassette drop-out flanked by connector sequences	ACTGATGAGACGTGGTAGAGCCACAAACAGCCGGTACAAGC<br>AACGATCTCCAGGACCATCTGAATCATGCGCGGATGACACGA<br>ACTCACGACGGCGATCACAGACATTAACCCACAGTACAGACA<br>CTGCGACAACGTGGCAATTCGTCGCAATACAACGTGAGACC<br>GAAAGTGAAACGTGATTTCATGCGTCATTTTGAACATTTTGTA<br>AATCTTATTTAATAATGTGTGCGGCAATTCACATTTAATTTATG<br>AATGTTTTCTTAACATCGCGGCAACTCAAGAAACGGCAGGTT<br>CGGATCTTAGCTACTAGAGAAAGAGGAGAAATACTAGATGCG<br>TAAAGGCGAAGAGCTGTTCACTGGTGTCGTCCCTATTCTGGT<br>GGAACTGGATGGTGATGTCAACGGTCATAAGTTTTCCGTGCG<br>TGGCGAGGGTGAAGGTGACGCAACTAATGGTAAACTGACGC<br>TGAAGTTCATCTGTACTACTGGTAAACTGCCGGTTCCTTGGC<br>CGACTCTGGTAACGACGCTGACTTATGGTGTTCAGTGCTTTG<br>CTCGTTATCCGGACCATATGAAGCAGCATGACTTCTTCAAGT<br>CCGCCATGCCGGAAGGCTATGTGCAGGAACGCACGATTTCC<br>TTTAAGGATGACGGCACGTACAAAACGCGTGCGGAAGTGAA<br>ATTTGAAGGCGATACCCTGGTAAACCGCATTGAGCTGAAAGG<br>CATTGACTTTAAAGAGGACGGCAATATCCTGGGCCATAAGCT<br>GGAATACAATTTTAACAGCCACAATGTTTACATCACCGCCGAT<br>AAACAAAAAAATGGCATTAAAGCGAATTTTAAAATTCGCCATA<br>ACGTTGAGGATGGCAGCGTGCAGCTGGCTGATCACTACCAG<br>CAAAACACTCCAATCGGTGATGGTCCTGTTCTGCTGCCAGAC<br>AATCACTATCTGAGCACGCAAAGCGTTCTGTCTAAAGATCCG<br>AACGAGAAACGCGATCATATGGTTCTGCTGGAGTTCGTAACC<br>GCAGCGGGCATCACGCATGGTATGGATGAACTGTACAAATG<br>ACCAGGCATCAAATAAAACGAAAGGCTCAGTCGAAAGACTGG<br>GCCTTTCGTTTTATCTGTTGTTTGTCGGTGAACGCTCTCTACT<br>AGAGTCACACTGGCTCACCTTCGGGTGGGCCTTTCTGCGTTT<br>ATAGGTCTCAGCTGGAAATCTGCTCGTCAGTGGTGCTCACAC<br>TGACGAATCATGTACAGATCATACCGATGACTGCCTGGCGAC<br>TCACAACTAAGCAAGACAGCCGGAACCAGCGCCGGCGAACA

		CCACTGCATATATGGCATATCACAACAGTCCACGTCTCAAGC AGTTACAGAGATGTTACGAACCG

Table S2: List of OCS constructs used

Name	Description	Transcriptional Units
OCS 1-1 (13,694bp)	U6 driven constitutive expression of gRNA-1 leading to constitutive expression of YFP under the control of pATF-1 promoter	TU1 : U6 :gRNA-1 (1013 bp)<br>TU2 : pATF-1: YFP: T35S (1231 bp)<br>TU3E : P35S : dCas9:VP64: T35S (5162 bp)
OCS 1-5 (13,685bp)	35S driven expression of gRNA-1 (flanked by ribozymes) leading to constitutive expression of YFP under the control of pATF-1 promoter	TU1 : P35S: HHR-gRNA-1-HDV:T35S (1001 bp)<br>TU2 : pATF-1 : YFP: T35S (1231 bp)<br>TU3E : P35S : dCas9:VP64: T35S (5162 bp)
OCS 1-9 (13,592bp)	Ethylene inducible expression of gRNA-1 under the control of EBS promoter. YFP under the control of pATF-1 promoter	TU1 : EBS: HHR-gRNA-1-HDV:T35S (917 bp)<br>TU2 : pATF-1 : YFP: T35S (1231 bp)<br>TU3E : P35S : dCas9:VP64: T35S (5162 bp)
OCS 4-1 (14,627bp)	U6 driven constitutive expression of gRNA-1 leading to constitutive expression of Luc under the control of pATF-1 promoter	TU1 : U6 :gRNA-1 (1013 bp)<br>TU2 : pATF-1 : Luc: T35S (2150 bp)<br>TU3E : P35S : dCas9:VP64: T35S (5162 bp)
OCS 3-5 (16,989bp)	Ratiometric circuit where YFP under the control of pATF-1 inducible by ethylene, while RFP and BFP under the control of Patf-3 are constitutively expressed	TU1 : EBS: HHR-gRNA-1-HDV:T35S (917 bp)<br>TU2 : pATF-1 : YFP: T35S (1231 bp)<br>TU3: pATF-3 : BFP : T35S (1228 bp)<br>TU4: U6 :gRNA3 (1013 bp)<br>TU5: pATF-3 : RFP : T35S (1214 bp)<br>TU6E : P35S : dCas9:VP64: T35S (5162 bp)

Table S3: List of Addgene plasmids used in this study

Name	Description
pICH86966<br>Addgene #48075	Used as the backbone of the shuttle vector; Contains the pVS1 and pMB1 replicons, KanR (bacterial) as well as a plant KanR under pNos.
pYTK001<br>Addgene #65108	Backbone for cloning all the parts listed in Table S1
pYTK002<br>Addgene #65109	Contains the connector LS; Used for the construction of TU1
pYTK003<br>Addgene #65110	Contains the connector L1; Used for the construction of TU2
pYTK004<br>Addgene #65111	Contains the connector L2; Used for the construction of TU3
pYTK005<br>Addgene #65112	Contains the connector L3; Used for the construction of TU4
pYTK006<br>Addgene #65113	Contains the connector L4; Used for the construction of TU5
pYTK007<br>Addgene #65114	Contains the connector L5; Used for the construction of TU6
pYTK067<br>Addgene #65174	Contains the connector R1; Used for the construction of TU1
pYTK068<br>Addgene #65175	Contains the connector R2; Used for the construction of TU2
pYTK069<br>Addgene #65176	Contains the connector R3; Used for the construction of TU3
pYTK070<br>Addgene #65177	Contains the connector R4; Used for the construction of TU4

pYTK071<br>Addgene #65178	Contains the connector R5; Used for the construction of TU5
pYTK072<br>Addgene #65179	Contains the connector RE; Used for the construction of TU6
pYTK095<br>Addgene #65202	Used as the backbone for the construction of any transcriptional unit.
